# Application of Combination Forecasting Model in Aircraft Failure Rate Forecasting

**DOI:** 10.1155/2022/6729608

**Published:** 2022-09-19

**Authors:** WenQiang Li, Chang Zhang

**Affiliations:** School of Mechatronics Engineering, Shenyang Aerospace University, Shenyang 110136, China

## Abstract

Effective prediction of aircraft failure rate has important guiding significance for formulating reasonable maintenance plans, carrying out reliable maintenance activities, improving health management levels, and ensuring the safety of aircraft flight, etc. Firstly, combining the advantages of time series model in eliminating random accidental factors interference, grey model in dealing with poor information, and the characteristics of artificial neural network in dealing with nonlinear data, the failure rate of aircraft equipment is predicted by ARIMA model, grey Verhulst model, and BP neural network model, and secondly, based on the idea of variable weight, the method of sum of squares of errors is used to reciprocate. Shapley value method and IOWA operator method determine the weighting coefficient and establish three combined forecasting models for aircraft failure rate prediction, so as to improve the accuracy of the algorithm. Finally, taking the data of actual aircraft failure rate as the research object, the performance indexes of design prediction model are judged by Mean Absolute Percentage Error (MAPE), Root Mean Square Error (RMSE), Mean Absolute Error (MAE), Index of Agreement (IA), Theil Inequality Coefficient (TIC), Equal Coefficient (EC), Nash-Sutcliffe Efficiency coefficient (NSE), Pearson test, and violin diagram of forecast error distribution. The experimental results show that: The forecasting precision of the combination model is better than that of the single model, and the evaluation index of combination forecasting model based on IOWA operator is better than that of other combination forecasting models, thus improving the forecasting accuracy and reliability. Compared with other typical prediction models simultaneously, it is verified that the proposed combined prediction model has strong applicability, high accuracy, and good stability, which provides a practical and effective technical method for aircraft fault prediction and has good application value.

## 1. Introduction

Aircraft, as a typical complex equipment, plays an important role in military and civil fields. Aircraft system is composed of many subsystems and related equipment. If any subsystem or equipment fails, it will seriously affect the flight performance and normal flight state of the aircraft, and will lead to corresponding safety accidents. Research on abnormal detection, fault diagnosis, and prediction technology of the aircraft system becomes the key to ensure the safe flight and efficient use of the aircraft. At this stage, most of the fault diagnosis uses post-event maintenance treatment, which has the disadvantages of poor real-time, long maintenance cycle, and large losses. It cannot meet the requirements of active prevention of aircraft failure and efficient configuration and management of maintenance resources. With the development of technology, the aircraft integrated support system is gradually developing from post-event maintenance to situation-based maintenance, fault prediction, and health management, which can significantly reduce the overall maintenance cost of aircraft, effectively reduce the probability of failure, and significantly improve the quality and efficiency of aircraft maintenance. The forecasting technology developed in 1960s has been widely used in many fields such as society, science and technology, military, etc. Scientific forecasting is the precondition and basis for correct decision-making and becomes an indispensable part of management and decision-making. Fault prediction involves key contents such as failure rate prediction, failure time prediction, performance prediction, life tracking, health assessment, spare parts management and maintenance decision, etc. Aircraft failure rate is one of the most important indicators to characterize the health status of aircraft, and is an important parameter for reliability-maintainability-supportability of aviation equipment as well as an important basis for guiding spare parts reserve. Forecasting aircraft failure rate scientifically can make scientific decision for aviation maintenance and is an indispensable important condition for improving maintenance support ability. It plays a very important role in improving the foreseeability, countermeasure, and scientificalness of aviation maintenance and guarantee work, as well as improving the perfection and utilization level of aviation equipment. Meanwhile, aircraft failure rate prediction technology has strong application value and broad development prospect. Therefore, failure rate prediction has become the focus of attention of many researchers. Because of the complexity of aircraft systems and the characteristics of randomicity, small sample size, and nonlinearity of failure rate, it is a great challenge to establish a predictive model of aircraft failure rate with satisfactory accuracy in the field of aeronautics.

At present, many scholars have put forward various methods and models for aircraft failure rate prediction and applied them in practice. Some scholars point out that the combination forecasting model has better forecasting effect on aircraft failure rate than the single forecasting model. However, the current research on portfolio forecasting model still has the following shortcomings. Firstly, the combination model formed by the single model is not comprehensive and systematic enough to analyze its overall impact during the construction process, and fails to fully consider the respective advantages of the models, and cannot fully exert the advantages of each model, resulting in the poor forecast effect of the combination model. Secondly, most of the weight coefficients of the combined forecasting model are solved by mean method, and the results are directly superimposed, ignoring the different effects and roles of different individual forecasting models in the whole combined model. Thirdly, the forecasting accuracy and efficiency of combined forecasting model are not high, and its applicability is poor. However, problems still need to be fixed. In order to improve the accuracy, efficiency, stability, and reliability of fault rate prediction, this paper will carry out corresponding research on the above deficiencies of existing combined forecasting models. The rest of this paper is as follows: Section 2 summarizes and analyzes the literature on aircraft failure rate prediction technology which has become popular in recent years.  Section 3 presents the combined forecasting method for aircraft failure rate, which is studied from the aspects of combined model modeling process, single model analysis, multi-combination model construction for solving variable weight coefficient, and model evaluation indexes.  Section 4 uses various models to study the aircraft failure rate with specific examples.  Section 5 compares and analyzes the forecast results of the model. The sixth section analyzes and discusses various models, and the seventh section gives a summary of this paper and relevant suggestions.

## 2. Literature Review of Aircraft Failure Rate Prediction

At present, there are many aircraft failure rate prediction models with different prediction effects, which can be roughly divided into single prediction model and combined prediction model. The aircraft failure rate prediction method is shown in Table 1.

Single prediction model can be divided into four types, including statistical model, grey model, machine learning model, and deep learning model. The statistical model is based on strict statistical theory and historical data information, which is used to extract the correlation between relevant variables or explanatory variables, and to establish and predict the model by statistical methods. Statistical models include regression analysis model [1], time series model (ARMA [2], SARIMA [3]), mathematical statistics model [4], Weibull statistical distribution model [5], Bayesian model [6], etc. The statistical model is characterized by a physical model to find the mapping relationship between the current state and future faults. The regression model has simple structure, wide calculation and application, but the accuracy is not high. Although the time series model has some related problems, such as the difficulty in parameter estimation of the high-order model and the low-order prediction accuracy, it has certain advantages in convenient calculation, providing linear smooth prediction, and excluding the interference of random accidental factors. The mathematical statistics model is affected by many other factors, and the overall forecast fluctuates greatly. The Weibull statistical distribution model has greater applicability than the logarithmic normal distribution, but the analytical estimation of Weibull distribution parameters is complex and the interval estimation is too long, thus reducing the prediction accuracy. Bayesian model has a good performance in predicting small-scale data, which is not sensitive to missing data and the algorithm is relatively simple. However, the prediction effect is not good due to the uncertainty of the prior model. At the same time, the above statistical model assumes that there is a linear relationship between aircraft failure rate and external influencing factors, which cannot be effectively applied to the complex nonlinear process of aircraft failure rate prediction. Therefore, there will be some poor performance of prediction results, which cannot achieve the ideal prediction effect.

Grey model includes grey GM (1, 1) model [7, 8], GM (1, 1) improved model [9], grey Verhulst model [10], etc. The grey model can be used to model and predict the aircraft system failure by the method of grey failure rate according to the characteristics of less source sample data. However, the grey model cannot be applied to practical applications such as large historical fault information data, large random fluctuation, and long-term prediction. Grey Verhulst model requires less data and convenient calculation. Since the aircraft fault is generally a small sample event and the amount of fault data is small, the grey Verhulst model has certain advantages in predicting the failure rate.

With the development of technology, artificial intelligence models have been applied to aircraft failure rate prediction, which usually includes machine learning and deep learning modeling methods. Machine learning includes artificial neural network (ANN) model [11], BP artificial neural network model [12, 13], fuzzy BP neural network [14], generalized regression neural network (GRNN) model [15], and other intelligent models, which have been used for accurate aircraft failure rate prediction. However, the neural network model has some shortcomings, such as difficult in scientifically determining the network structure, slow learning speed, existence of local optimal value, and memory instability, which makes the prediction accuracy difficult to guarantee. At the same time, the neural network needs a large number of sample data, which increases the calculation and prediction time. According to statistics, most aircraft failure rate data have nonlinear characteristics. BP neural network is widely used in failure rate prediction because it can deal with nonlinear data well and can effectively improve prediction accuracy. At the same time, the support vector machine (SVM) [16] model and least squares support vector machine (LSSVM) [17] model are also applied to the prediction of aircraft failure rate and achieved certain prediction effects and accuracy. The advantages of the SVM model and least squares support vector machine model are that fewer samples are needed and nonlinear correlation data can be processed. But they have shortcomings in that model parameters are difficult to determine. Because the random forest method [18] can deal with classification and regression problems well, it is also applied to the field of aircraft failure rate prediction. Machine learning method can effectively improve the prediction accuracy of aircraft failure rate, so it is widely used. However, there are also shortcomings. For example, the learning speed is not ideal, and a large amount of data is needed for training and learning. The complexity of the algorithm is increased and the real-time performance of the prediction model is affected.

In recent years, with the mature development and application of deep learning methods such as Long short-term memory (LSTM) neural network technology [19] and convolution neural network (CNN) technology [20], some researchers have conducted valuable research in the field of aircraft failure rate prediction because of its advantages in data feature extraction. However, the deep learning model has theoretical limitations, resulting in many deficiencies in practical applications, such as large training samples, time-consuming, complex structure, difficult to determine its structural parameters, and premature convergence. These deficiencies will affect the use of deep learning model and make it unable to achieve good prediction results. The effect of deep learning model in small sample prediction is even worse than that of traditional machine learning and grey prediction model.

Although these single prediction models and methods have achieved good results, they have their own shortcomings and limitations. These single prediction models have their unique information characteristics and applicable conditions, which can only reflect the future situation of failure rate from different aspects. Since the fault of aircraft system has certain randomness, complexity, and uncertainty, the prediction results of single prediction model often cannot fully reflect the failure rate and some prediction accuracy is not high. Single prediction model usually only contains part of the information of the prediction object. However, combining various single models by using certain rules, it can contain more comprehensive prediction information to improve the prediction accuracy. Therefore, some scholars have proposed a combination model. Combination forecasting is an important research branch in the field of forecasting. Since Bates and Granger first proposed the combination forecasting theory system in 1969, this method has been widely concerned by scholars at home and abroad. Effective combination of different prediction models can be regarded as an effective supplement to the generation process of infinitely approaching real data. Combination forecasting method is complementary to the advantages of single model, which can combine the advantages of various single models, so as to effectively improve the prediction accuracy of the model. It is a hot research topic in recent years.

Combination forecasting models can generally be divided into model-based combination, method-based combination, and decomposition-based combination models. Model-based combination model refers to a new model composed of multiple single models, forming a combination model, generally composed of 2–6 single models, or more models, but the prediction effect of the combination model will not improve obviously with the increase of the number of models. Model-based combinatorial models have been studied in recent years, and there are many combinatorial models and methods. For example, the [21] grey neural network and fuzzy recognition model are proposed to realize the fault prediction of avionics system, and the accuracy of the algorithm is improved by this method. Combining artificial neural network with genetic algorithm [22], it proposed constructing a combined prediction model of hybrid single model by analyzing the factors affecting the failure rate of airborne equipment based on [23] optimal combination forecast model, and the prediction performance of the combined model is verified by experiments. A combined model of [24] support vector regression (SVR), multiple regression, and principal component analysis is proposed. Establishing a mathematical relationship between aircraft failure rate and its complex influencing factors and testing the proposed method by using the statistical data of aviation equipment quality control, the prediction results show the effectiveness of the proposed method [25]. ARMA-BP combination model [26], grey model and neural network combination model [27], grey multiple linear regression fusion model, and many combination forecasting models are proposed. The above combined prediction method has achieved a certain prediction effect on aircraft failure rate prediction and improved the accuracy of some predictions. However, due to the complexity of the combined model, it is difficult to optimize the parameters of the single model, cannot give full play to the advantages of each single model, and the weight is difficult to determine, which needs to be further studied to gradually improve the prediction effect.

Method-based combination model is to combine certain methods into single model to predict aircraft failure rate and improve the prediction performance of the model. Applying this optimization method to aircraft failure rate prediction can improve the prediction performance of the original model. It includes Holt-Winters seasonal model [28], AR model of neural network residual correction [29], Weibull regression model of artificial neural network [30], Weibull-based Generalized Renewal Process (WGRP) [31], Sparse direct support vector regression machine [32], Generalized weighting least-squares combination prediction [33], and other models to predict the failure rate, which has certain prediction effect. However, the structure and parameters of the combined model are uncertain in the prediction, and different parameters and structures will have a great impact on the prediction structure. It needs to be further verified by optimized and selected parameters and structures, and the optimal parameters and structures are used for prediction to improve the prediction performance of the model.

The combination model based on decomposition ensemble method generates different characteristic components and lets them be predicted by the same or different models by decomposing the original data. Finally, the predicted values of each component are superimposed and integrated to form the final predicted values. These decomposition ensemble methods include empirical mode decomposition and LS-SVM combination [34], correlation vector EMD and GMDH reconstruction combination [35], EMD and RVM-GM model [36], CEEMD and combinatorial model [37], and other prediction models. These methods can decompose the original aircraft failure rate data into many components with different characteristics, and then use the appropriate prediction model to predict each component. Finally, the final prediction value is obtained by reconstruction and integration, which reduces the aircraft failure rate data including noise, random fluctuation, and other factors. Many scholars have carried out various studies in this area, and this method was used to conduct experiments and applications in the failure rate prediction field. The above combined models make full use of the advantages of various methods and models, and have achieved good prediction results. The combined prediction method has become a mainstream direction of aircraft failure rate prediction in recent years. But, through the analysis of the combination forecasting model, the current combination forecasting still has the following problems.

1. The selection of methods and quantities of single forecasting model participating in combination: Since each single forecasting model has its applicable conditions, it is generally necessary to give full play to the advantages of single models and avoid their shortcomings when selecting single models to establish the combination models. However, there is no suitable selection principle on how to select suitable single models for prediction objects. At the same time, there is also uncertainty in the selection of the number of single forecasting models. It is generally believed that the prediction performance of the combined prediction is improved with the increase of the number of single forecasting models. However, too many models will increase the complexity, and the actual prediction accuracy will decrease. Therefore, how to select the appropriate number of single forecasting models needs further research.
2. The selection of weighting methods: most of the current combined forecasting models use the time-invariant weighting model, that is, the weight coefficients of the same single forecasting method in each period are the same. But actually, the prediction accuracy of the same single forecasting method in different periods is different, which is manifested in the high prediction accuracy in a certain period, and the low prediction accuracy in another period. Solving weights by different methods has a great influence on the accuracy and prediction performance of the combined forecasting model.
3. Applicability of Combination Forecasting Model: Each prediction model should have certain adaptability. However, when establishing the combined prediction model, most of the models are constructed under given assumptions, and there is no corresponding limitation on the solution method of the mathematical model. Different models are suitable for different occasions, so the different application occasions of the combined model also have a great impact on the prediction accuracy.

The problems existing in the abovementioned combined models are the difficult problems in the current research on combined prediction. Because the aircraft system has the characteristics of small batch, multivarieties, complex system cross-linking, and random faults. In addition, the failure rate information sample data source is less, the lack of effective fault characteristics, fault diversification, and the failure rate information has the characteristics of nonlinear change. Besides, the aircraft failure rate is also affected by random interference factors such as weather conditions, sudden state, technology, and management level. Therefore, the selection of high-precision and efficient failure rate prediction models and modeling methods are still a hot research topic. Given this, this paper will study the aircraft failure rate prediction method, and put forward the combination model to predict the aircraft failure rate to improve the accuracy and quality of aircraft failure rate prediction. In the combined model developed in this study, first of all, three models in statistical model, grey model, and machine learning method, namely, ARIMA model, grey Verhulst model, and BP neural network model, are used to effectively predict the failure rate of an aircraft. It gives to play the advantages of combining the time series model to eliminate the interference of random accidental factors, the grey model to deal with poor information, and the artificial neural network to deal with nonlinear data, so as to give full play to the advantages of each model. Secondly, on the basis of not increasing the complexity, three combination forecasting models are constructed based on the variable weight idea by solving the weight coefficient with error sum of squares reciprocal method, Shapley value method, and IOWA operator method. Finally, the effectiveness and applicability of the proposed combination forecasting model in aircraft failure rate prediction are verified by examples, which provide an effective basis and foundation for aircraft fault diagnosis and health management.

## 3. Combination Forecast Model of Aircraft Failure Rate

**Figure 1:**
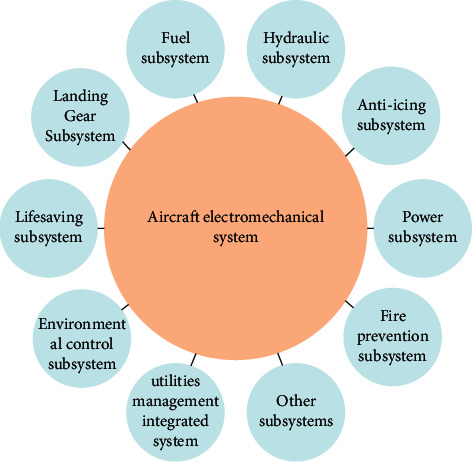
Composition of aircraft electromechanical system.

The electromechanical system is one of the core key systems of the aircraft. Ensuring the stable operation of the system can effectively improve the safety and reliability of the aircraft. The electromechanical system mainly consists of fuel subsystem, hydraulic subsystem, landing rack system, life-saving subsystem, and other subsystems, as shown in Figure 1. As the mechanical, electrical, hydraulic, and control circuit components involved in the electromechanical system of the aircraft are organically combined, the composition structure is relatively complex. With the increase of service time, it is prone to failure, which will lead to the failure of the aircraft to complete the specified functions, thus causing serious damage to the aircraft. Moreover, because of resource interweaving, system resource sharing, and high functional coupling, the fault propagation path of electromechanical system presents multidimensional and complex characteristics, which brings great difficulties to fault rate diagnosis and prediction. Considering the strong disturbance from random accidental factors, the lack of information, and the nonlinear relationship between historical data of aircraft electromechanical system. In this paper, the time series method with strong resistance to accidental factors, the grey theory applicable to small samples, and the neural network algorithm with strong nonlinear mapping ability are combined, and a combined prediction model is proposed and applied to the prediction of aircraft failure rate. It is of great significance and value to improve the health state estimation performance of the aircraft electromechanical system through the research on the failure rate prediction of the aircraft electromechanical system, so as to promote the technical development in the field of flight mission support, condition-based maintenance, and health management of the aircraft electromechanical system. This paper will take the failure rate of aircraft electromechanical system as an example to study the system. The failure rate of aircraft described in this paper refers to the failure rate of aircraft electromechanical system.

### 3.1. Process of Combined Model Modeling

**Figure 2:**
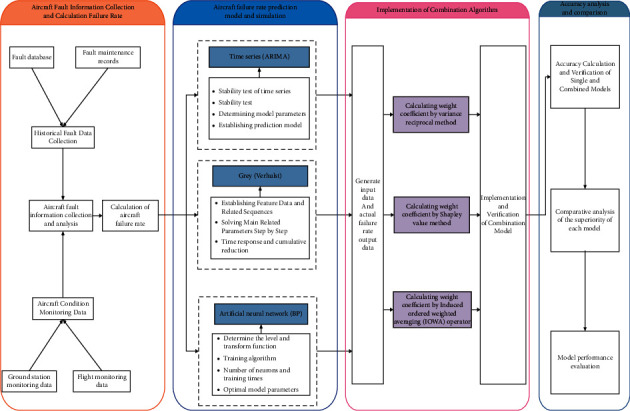
Flow chart of composite model modeling.

In order to study the aircraft failure rate prediction model, the aircraft failure rate is predicted based on the single time series ARIMA model, grey Verhulst model, and BP artificial neural network model. On this basis, the weight coefficient is solved by the inverse of error square sum method, Shapley value method, and IOWA operator method, so as to form different combined prediction models, analyze and study them, and compare and evaluate the performance of different models. The modeling process of the combined model is shown in Figure 2, which specifically includes the following four parts: first, collect aircraft fault information and calculate the fault rate, analyze the fault situation of an aircraft from 2012 to 2018, collect fault data from the historical fault database and fault maintenance records of the maintenance management system, and complete the collection of historical fault data. The data obtained from the two aspects are used for analysis and screening, and then the failure rate of the aircraft is calculated, and the average failure rate is taken as the overall failure rate. Secondly, three single models are used to model and simulate the failure rate. Based on the failure rate data obtained in the previous step, three single models and related parameters based on time series ARIMA model, grey Verhulst model, and BP neural network model are constructed. The historical data of the aircraft failure rate of the three single prediction models are used as training input, and the subsequent predicted failure rate value is used as the dependent output variable of the single model for research. Thirdly, combined model construction and research are carried out. In this stage, based on the predicted value of single model failure rate, three combined prediction models are built based on the inverse of error square sum method, Shapley value method, and Iowa algorithm method to solve the weighting coefficient, which provides a variety of more effective models for accurate prediction of aircraft failure rate. Finally, the accuracy of different models is analyzed and compared, and the results of single model and the three combined prediction models are compared. A variety of indicators are used as evaluation criteria to analyze, discuss their advantages and evaluate the performance of various models.

### 3.2. Single Model

#### 3.2.1. ARIMA Model

In 1970, the American scholar box first proposed the classical analysis theory, modeling, and prediction method of time series [38]. The integrated moving average autoregressive model (ARIMA) is one of the commonly used time series and has been widely used in the prediction fields of aviation, aerospace, and engineering:

(1) $$\begin{array}{c} \Phi \left(L\right){\left(1-L\right)}^{d}y_{t}=\varepsilon +\Theta \left(L\right)\varepsilon_{t}\text{,} \end{array}$$

In which, Φ(*L*)=1 − *λ*_1_*L* − *λ*_2_*L* − ⋯−*λ*_*P*_*L*^*P*^ is the polynomial of *P*-order autoregressive coefficient, Θ(*L*)=1+*θ*_1_*L*+*θ*_2_*L*+⋯+*λ*_*q*_*L*^*q*^ is the polynomial of *P*-order moving average coefficient, *L* is the lag operator, *λ* and *θ* are estimated values of each independent variable, respectively, *y*_*t*_=*c*+*y*_*t*−1_+*μ*_*t*_ is *D*-order monointeger sequence, *c* is constant, *μ*_*t*_ is stationary sequence, *t*=1,2,…, *T*; *ε*_*t*_ is the white noise order with mean value of 0 and variance of *σ*^2^.

The prediction steps of ARIMA model are: Step 1: stationarity and testing; Step 2: smoothing processing; Step 3: model identification and order determination; Step 4: model parameter estimation; Step 5: model test; Step 6: model prediction.

**Figure 3:**
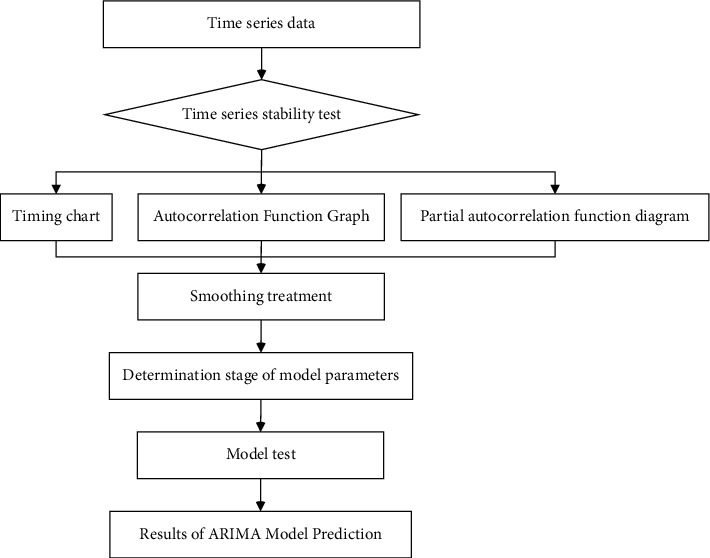
Flow chart of ARIMA modeling.

The modeling process is shown in Figure 3:

#### 3.2.2. Grey Verhulst Model

The grey system theory was first proposed by Deng et al. to deal with the system of “small samples and poor information.” The grey system theory takes small samples with known and unknown information, poor information, and uncertain systems as the research object, which makes up for the inadequacy of statistical analysis methods. The grey Verhulst model is a component of the grey system theory, which has been widely used in prediction [39]. The prediction steps of grey Verhulst model are as follows: Step 1: Carry out accumulation generation operation; Step 2: Generate a sequence of immediate mean values; Step 3: Establish approximate time response sequence; Step 4: Prediction model of progressive reduction.

**Figure 4:**
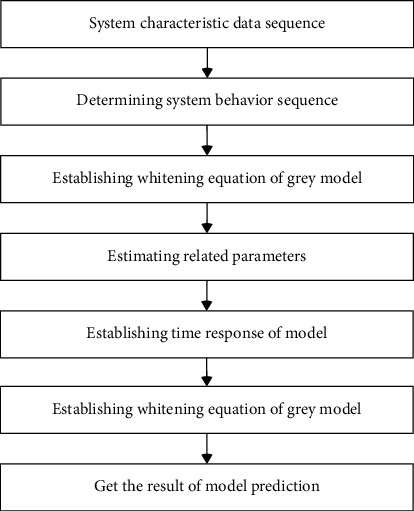
Grey Verhulst modeling flow chart.

The grey Verhulst modeling flow chart is shown in Figure 4.

#### 3.2.3. BP Artificial Neural Network Model

In 1985, Rumelhart proposed the BP algorithm. As the most widely used artificial neural network, BP neural network is a multi-layer feedforward neural network. This model is widely used in the prediction field [40]. The training process of BP neural network includes the following seven steps: Step 1: network initialization; determining the number of nodes *n* of the input layer, the number of nodes *h* of the hidden layer, and the number of nodes *m* of the output layer according to the input and output sequences of the system; initializing the connection weights between the neurons of the input layer, the hidden layer, and the output layer; initializing the threshold of the hidden layer and the threshold of the output layer; and giving the learning rate and the neuron excitation function; Step 2: hidden layer output calculation; Step 3: output calculation of output layer: calculate the predicted output of BP neural network according to the output of hidden layer, connecting the weight and threshold. Step 4: error calculation. Step 5: weight update; Step 6: threshold update; Step 7: judge whether the algorithm iteration is finished. If not, return to Step 2 and perform calculation.

**Figure 5:**
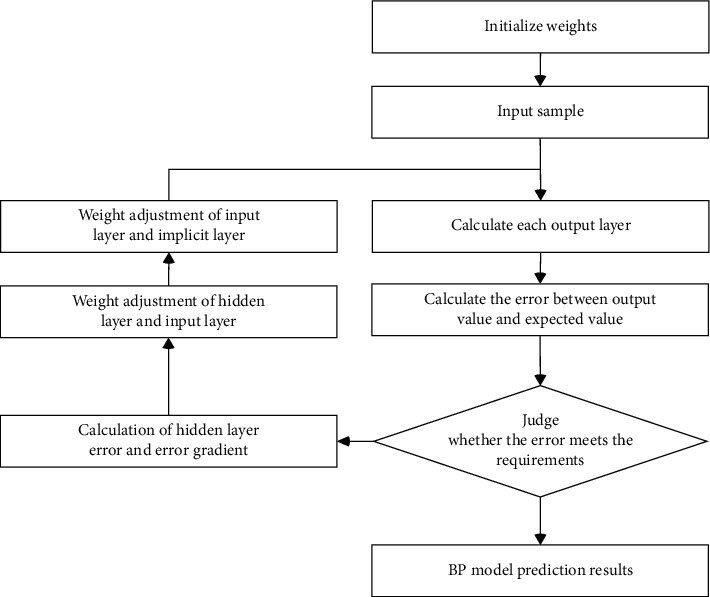
BP neural network algorithm flow.

The algorithm flow of BP neural network is shown in Figure 5.

### 3.3. Combination Forecasting Model Based on Variable Weight

In most cases, the single model will have some shortcomings. Only by extracting the advantages of each single model and combining them can the advantages of each single model be brought into play to form an optimal combined prediction model.

#### 3.3.1. The Combined Forecasting Model Based on the Reciprocal of Error Sum of Squares

The inverse of the square sum of prediction error method first needs to calculate the square sum of the error between the predicted value and the real value. The smaller the calculated value indicates, the higher the accuracy of the prediction, the greater the weight of the model in the combined model, and vice versa. Let *ω*_*i*_ be weight coefficient, *i*=1,2,…*n*; calculate the sum of error squares of each single prediction model *e*_*i*_, *i*=1,2,…*n*; then, give a larger weight to the model with a smaller sum of squares of errors, and give a smaller weight to the model with a larger sum of squares of errors. Through calculation, the weight coefficient is *ω*_*i*_=*e*_*i*_^−1^/∑_*i*=1_^*n*^*e*_*i*_^−1^, so as to obtain the final combined forecasting model.

#### 3.3.2. Combined Forecasting Model Based on Shapley Value

It is assumed that there are *n* kinds of prediction methods for combined prediction, which is denoted as *I*={1,2,…*n*}. For any subset *s* of *I*, *E*(*s*) represents the error of each combination. Let the absolute mean of the prediction errors of the *i* prediction methods be *E*_*i*_, and the total error of the combined prediction be *E*.

(2) $$\begin{array}{c} E_{i}=\frac{1}{m}\overset{m}{\underset{j=1}{\sum }}\left|e_{ij}\right|,i=1,2,\ldots n;\;E=\frac{1}{n}\overset{n}{\underset{j=1}{\sum }}E_{i}\text{.} \end{array}$$

Shapley value error distribution formula is:

(3) $$\begin{array}{c} E_{i}^{/}=\sum_{s\in i}\omega \left(s\right)*\left[E\left\{s\right\}-E\left(\left\{s\right\}-\left\{i\right\}\right)\right],i=1,2,3,\ldots n;\;W\left(\left|s\right|\right)\frac{\left(n-\left|s\right|!\right)\left(\left|s\right|-1\right)}{n!}\text{.} \end{array}$$

In which, *i* represents the *i*th prediction model in the combination, *E*_*i*_^/^ represents the Shapley value of the ith prediction model, that is, the allocated error, *s* represents the combination including the prediction model, |*s*| represents the number of prediction models in the combination, and *W*(|*s*|) can be regarded as a weight, which is the weighting factor of the combination prediction. The weight calculation formula: *ω*_*i*_=1/*n* − 1*E* − *E*_*i*_^/^/*E*, *i*=1,2,…, *n* can be obtained from the above, and the corresponding combination prediction model can be obtained.

#### 3.3.3. Combination Forecasting Model Based on IOWA Operator

Suppose there are *n* kinds of forecasting methods for forecasting. Let *y*_*t*_ denote the actual observation value at time *t*, *y*_*it*_ represents the predicted value of method *i* at time *t*, *e*_*it*_ represents the prediction error of method *i* at time *t*, (*e*_*it*_=*y*_*t*_ − *y*_*it*_, *i*=1,2,…, *n*; *t*=1,2,…, *T*), *ω*_*i*_ represents the weight of method *i* in the combined prediction model, (*i*=1,2, ⋯, *n*; ∑_*i*=1_^*n*^*ω*_*i*_=1), Then the calculation formulas of prediction value and error in sample period are ${\hat{y}}_{t}=\sum_{i=1}^{n}\omega_{i}y_{it}$, The prediction accuracy is used as the inducing factor. If the inducing factor *α*_*it*_(*i*=1,2,…, *n*; *t*=1,2,…, *T*) select the prediction accuracy which used the *i*-th prediction method in the *t*-th period. Then, the expression of *α*_*it*_ is:

(4) $$\begin{array}{c} \alpha_{it}=\left\{\begin{array}{cc} 1-\left|\frac{y_{t}-{\hat{y}}_{it}}{y_{t}}\right|\text{,} & \left|\frac{y_{t}-{\hat{y}}_{it}}{y_{t}}\right|\leq 1\text{,}\\ 0\text{,} & \left|\frac{y_{t}-{\hat{y}}_{it}}{y_{t}}\right|\geq 1\text{.} \end{array}\right. \end{array}$$

The *n* two-dimensional arrays generated by the inducing factors (〈*α*_1*t*_, *y*_1*t*_ > , <*α*_2*t*_, *y*_2*t*_ > ,…, <*α*_*nt*_, *y*_*nt*_〉) are arranged in descending order of the inducing factors. According to the minimum error square sum criterion, the weight coefficient vector of each precision is obtained as *W*=(*ω*_1_, *ω*_2_,…,*ω*_*n*_)^*T*^ and satisfies (∑_i=1_^*n*^*ω*_*i*_=1, *ω*_*i*_ ≥ 0, *i*=1,2,…, *n*). The *y*_1*t*_, *y*_2*t*_,…, *y*_*nt*_(*t*=1,2,…, *T*) is generated by prediction accuracy *α*_1*t*_, *α*_2*t*_,…, *α*_*nt*_, then the induced prediction error predicted by IOWA operator combination prediction model is *e*_*α*−in de x(*it*)_=*y*_*t*_ − *y*_*α*−in de x(*it*)_(*i*=1,2,…, *n*; *t*=1,2,…, *T*). Let *R*_*n*_=(1,1,…,1)^*T*^ be the m-dimensional unit vector, and the constraint condition of the weight vector *W*=(*ω*_1_, *ω*_2_,…,*ω*_*i*_)^*T*^ be *R*_*n*_^*T*^*W*=1, *W* ≥ 0. Therefore, the prediction error of IOWA combined prediction model at time *t* is:

(5) $$\begin{array}{c} y_{t}-{\hat{y}}_{t}=y_{t}-\overset{n}{\underset{i=1}{\sum }}\omega_{i}y_{a-in\;de\;x\left(it\right)}=\overset{n}{\underset{i=1}{\sum }}\omega_{i}e_{a-in\;de\;x\left(it\right)}\left(t=1,2,\ldots T\right)\text{.} \end{array}$$

The sum of squares of the total prediction errors of the model is:

(6) $$\begin{array}{c} Q={\overset{T}{\underset{t=1}{\sum }}\left(y_{t}-\overset{n}{\underset{i=1}{\sum }}\omega_{i}y_{a-in\;de\;x\left(it\right)}\right)}^{2}={\overset{T}{\underset{t=1}{\sum }}\left(\overset{n}{\underset{i=1}{\sum }}\omega_{i}e_{a-in\;de\;x\left(it\right)}\right)}^{2}=\overset{n}{\underset{i=1}{\sum }}\overset{n}{\underset{j=1}{\sum }}\omega_{i}\omega_{j}{\bar{E}}_{ij}\;is\;the\;polynomial\;of\;P\text{.} \end{array}$$

In which: ${\bar{E}}_{ij}={\bar{E}}_{ji}=\sum_{t=1}^{T}e_{a-in\;de\;x\left(it\right)}e_{a-in\;de\;x\left(jt\right)},i,j=1,2,\ldots ,n$. Let $\bar{E}={\left({\bar{E}}_{ij}\right)}_{n\times n}$ be the prediction error information matrix of the *n*-order IOWA, then $Q=W^{T}\bar{E}W$. Therefore, the IOWA combination prediction model based on the optimization criterion for minimum error sum of squares is:

(7) $$\begin{array}{c} \min\text{ ,}Q=W_{n}^{T}{\bar{E}}_{n}W_{n,}\\ s_{•}t_{•}\;\left\{\begin{array}{c} R_{n}^{T}W=1,\\ W\geq 0. \end{array}\right. \end{array}$$

### 3.4. Evaluation Index of Model

In order to more accurately evaluate the effectiveness of the prediction model and comprehensively display the performance indicators of the proposed model, seven evaluation indicators are adopted, including Mean Absolute Percentage Error (MAPE), Root Mean Square Error (RMSE) [41], Mean Absolute Error (MAE), Index of Agreement (IA), Theil Inequality Coefficient (TIC), Equal Coefficient (EC), and Nash-Sutcliffe Efficiency coefficient (NSE). The effectiveness of each prediction method is evaluated by using the above indexes.

(8) $$\begin{array}{c} MAPE=\frac{1}{n}\overset{n}{\underset{i=1}{\sum }}\left|\frac{{\hat{y}}_{i}-y_{i}}{y_{i}}\right|\times 100\%\text{,}\\ RMSE=\sqrt{\frac{1}{n}\overset{n}{\underset{i=1}{\sum }}{\left({\hat{y}}_{i}-y_{i}\right)}^{2}}\text{,}\\ MAE=\frac{1}{n}\overset{n}{\underset{i=1}{\sum }}\left|{\hat{y}}_{i}-y_{i}\right|\text{,}\\ IA=1-\frac{\sum_{i=1}^{n}{\left({\hat{y}}_{i}-y_{i}\right)}^{2}}{\sum_{i=1}^{n}{\left(\left|{\hat{y}}_{i}-\bar{y}\right|+\left|y_{i}-\bar{y}\right|\right)}^{2}}\text{,}\\ TIC=\frac{\sqrt{1/n\sum_{i=1}^{n}{\left(y_{i}-{\hat{y}}_{i}\right)}^{2}}}{\sqrt{1/n\sum_{i=1}^{n}y_{i}^{2}}+\sqrt{1/n\sum_{i=1}^{n}{\hat{y}}_{i}^{2}}}\text{,}\\ EC=1-\frac{\sqrt{\sum_{i=1}^{n}{\left(y_{i}-{\hat{y}}_{i}\right)}^{2}}}{\sqrt{\sum_{i=1}^{n}{\hat{y}}_{i}^{2}}+\sqrt{\sum_{i=1}^{n}y_{i}^{2}}}\text{,}\\ NSE=1-\frac{\sum_{i=1}^{n}{\left(y_{i}-{\hat{y}}_{i}\right)}^{2}}{\sum_{i=1}^{n}{\left(y_{i}-\bar{y}\right)}^{2}}\text{.} \end{array}$$

It is assumed that the predicted value is $\hat{y}=\left\{{\hat{y}}_{1},{\hat{y}}_{2},\ldots ,{\hat{y}}_{i}\right\}$, the real value is *y*={*y*_1_, *y*_2_,…, *y*_*i*_}, the average value of the real value is $\bar{y}$, and the average value of the predicted value is $\bar{\hat{y}}$. According to the definition of the above indicators, MAPE represents the average error of multiple prediction results. The smaller the value, the higher the prediction accuracy. Generally, when MAPE < 10, the prediction accuracy is considered to be better. It is used to check the deviation and fluctuation between the actual value and the predicted value. The closer the RMSE is to 0, the higher the accuracy of the prediction model. At the same time, the Mean Absolute Error (MAE) is small when the predicted value is in good agreement with the real value. The closer the value of IA (index of agreement) is to 1, the higher the change trend, consistency, and consistency between the predicted value and the actual value. The value of Theil Inequality Coefficient (TIC) is between [0, 1]. The closer to 0, the smaller the fitting error. The larger the value of EC (equal coefficient), the better the prediction effect of the model. Generally, 0.9 or more is considered as a good fit. The value range of Nash efficiency coefficient is (−*∞*, 1), and the closer it is to 1, the better the prediction quality and the higher the reliability.

## 4. Case Study of Each Model

**Figure 6:**
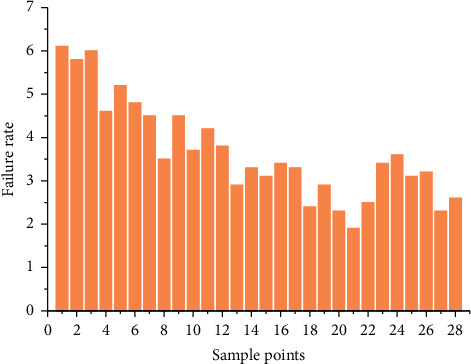
Data set of aircraft failure rate.

Taking a certain type of aircraft electromechanical system as the research object, the fault data of this electromechanical system are collected in various ways, and the corresponding failure rate is calculated, so as to carry out the research of single and combined prediction models. Different use environments, use methods, maintenance quality, random interference and other factors will affect the failure rate. However, some influencing factors are difficult to obtain in practice, and the quantification is not accurate, which affects the prediction accuracy. Therefore, this paper only considers the overall failure rate, without considering other influencing factors, and takes the average failure rate as the failure rate in the research. The average failure rate is the ratio of the total number of failures *n*_*f*_(*t*) of the system in a specified period of time to the cumulative working time *T*. The formula for calculating the failure rate is $\bar{\lambda }=n_{f}\left(t\right)/T$. Take the fault data of each quarter as one observation value, that is, take one quarter as the statistical interval every year to form four observation values, collect the fault data from 2012 to 2018, calculate the fault rate of these seven years, form a set of time series data, including 28 observation values, and then take 28 groups of basic data samples as examples for research. In order to measure the accuracy of different models and prevent overfitting, the collected data are divided into two sub sets: the test input data set (including 70% of the data) and the test data set (including 30% of the data). The sample data are shown in Figure 6. Select 1–20 samples of 20 quarters from 2012 to 2016, i.e., the first 20 groups of data, as the input of the model, and select 21–28 samples of working time distribution from 2017 to 2018, i.e., the last 8 groups of data, to test the prediction model. ARIMA model, grey Verhulst model, and BP neural network model are established, respectively. On the basis of single prediction model, variable weight combined prediction model based on the reciprocal of error square sum method, Shapley value method, and IOWA operator are established. Relevant models are established and studied. From the figure, it can be seen that the fault rate data have strong randomness, nonstationary characteristics, small number of samples, and certain nonlinear characteristics.

### 4.1. Analysis of ARIMA Model

The time series composed of the first 20 groups of data of samples 1–20 is used for data processing and programming. The stationarity of the data is tested by kPSS function and ADF function. When kSPP = 0 and ADF = 1, it indicates that the tested sequence is a stable sequence. The results after operation show that the original sequence needs to undergo the third-order difference before it can be converted into a stationary sequence. Therefore, the model is initially determined as ARIMA (*p*, 3, *q*) model. Autocorrelation function and partial autocorrelation function are trailed according to the AIC criterion and BIC criterion; when *p* = 2 and q = 2, the AIC and BIC values are relatively minimum, so the time series model is ARIMA (2, 3, 2). Thus, the prediction equation of ARIMA model is obtained, and then the last 8 groups of data are predicted to obtain the predicted values of the corresponding 8 groups of test data.

### 4.2. Analysis of Grey Verhulst Model

The first 20 sets of data of failure rate sample 1–20 are selected as independent variable time series to form original data series *X*^(1)^={*X*^(1)^(1), *X*^(1)^(2),…, *X*^(1)^(20)}, and generate *X*^(1)^ by one-time progressive subtraction (1-iAGO). Conduct modeling according to the steps described in section 3.2.2, and obtain the corresponding prediction model data *a* = 0.06, *B* = 0.01 by using the grey system modeling software (v7.0) of China Southern Airlines. The corresponding grey Verhulst model is: d*x*^(1)^/d*t*+0.06*x*^(1)^=0.01(*x*^(1)^)^2^, and its time response is ${\hat{x}}_{k+1}^{\left(1\right)}=ax_{1}^{\left(0\right)}/bx_{1}^{\left(0\right)}+\left(a-bx_{1}^{\left(0\right)}\right)e^{ak}=0.36/0.01+0.05e^{0.06k}$. Establishing the corresponding prediction model, so as to obtain the approximate time response formula, and the grey Verhulst prediction model is obtained through inverse accumulated generating operation (IAGO). Then, the last 8 groups of inspection data prediction values are obtained on this basis.

### 4.3. Analysis of BP Model

Aircraft failure rate samples have strong randomness, which will greatly affect the learning speed and prediction accuracy of the neural network. In order to speed up the learning speed of the neural network and improve the prediction accuracy of the neural network, it is necessary to preprocess the failure rate data sequence before processing the aircraft failure rate data with the neural network, and to normalize the input and output variables with the maximum-minimum method. Make it fall completely within the interval [−1, 1]. According to the relevant principle of BP neural network algorithm, under the condition of reasonable structure of BP neural network and proper weights of neural nodes, 3-layer neural network can approximate any continuous function, and all nonlinear mapping from input to output can be realized by fully learning 3-layer BP neural network. The data samples are fitted by 3-layer BP neural network model. Since the statistical failure rate sample is a single value, the 1–20 sets of data (20 quarters in 2012–2016) constitute a one-dimensional sequence, but the input data of BP neural network algorithm needs to be learned from a multi-dimensional sequence. Based on the four-quarter aircraft failure rate data as the prediction basis, the current four-quarter data are taken as the input value of the neural network in turn, and the last four quarters data as the target data of the network data. Rolling arrangement is carried out in this way to form training samples of neural network, so the input layer node is determined to be 5. If the aircraft failure rate is taken as the only output of BP neural network model, then the number of output nodes is 1. BP neural network model with 5 input nodes, 3 network layers, and 1 output node is constructed. 2*K* + 1 hidden layer node is determined according to the number of hidden layer nodes of [42] neural network. K is the number of inputs and 11 hidden layer nodes are selected. The fitting error of BP neural network process is set as 10^−5^, and 1–20 groups of training data are input into the network for training. After 65 iterations, the output error is less than the convergence error. Based on this algorithm, the predicted values of the last 8 groups of test data are obtained.

### 4.4. Solution of Algorithm Coefficients and Construction of Model

**Figure 7:**
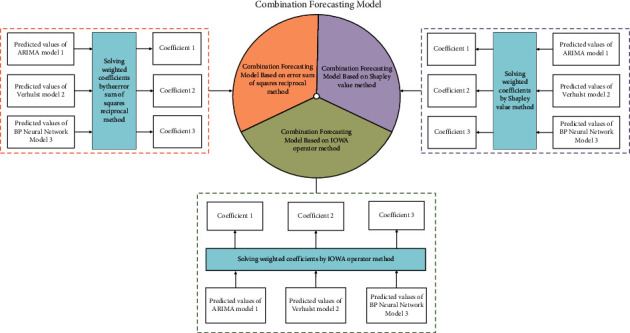
Graph of combination algorithm coefficient solution and construction.

The solution and construction process of the combined algorithm coefficients are shown in Figure 7.

ARIMA model, grey Verhulst model, and BP neural network model are used for single model prediction, and three groups of corresponding failure rate prediction values are obtained, respectively. Assuming that the predicted aircraft failure rate obtained by ARIMA model is ${\hat{y}}_{1t}$, the predicted aircraft failure rate obtained by grey Verhulst model is ${\hat{y}}_{2t}$, the predicted aircraft failure rate obtained by BP neural network model is ${\hat{y}}_{3t}$, the predicted value of aircraft failure rate obtained by the combined prediction model is ${\hat{y}}_{t}$, and *t* represents the corresponding time series from 1 to the forecast period. *ω*_1_, *ω*_2_, and *ω*_3_ are obtained by different weight coefficient solutions. Therefore, the expression of the combination model is: ${\hat{y}}_{t}=\omega_{1}{\hat{y}}_{1t}+\omega_{2}{\hat{y}}_{2t}+\omega_{3}{\hat{y}}_{3t}$. By substituting the data obtained from the single model into the formula, the predicted values of 8 groups of inspection data of the combined model solved by different weight coefficients are obtained.

#### 4.4.1. Combined Forecasting Model Based on the Error Sum of Squares Reciprocal Method

Based on the prediction data of ARIMA model, grey Verhulst model, and BP neural network model, the corresponding coefficients were solved according to the error sum of squares reciprocal (ESSR) method, and the corresponding weight coefficient values were obtained. Then, substituting the data obtained by the single models, the prediction values of 8 groups of aircraft failure rate inspection data of the combined prediction model based on the error sum of squares reciprocal method are obtained.

#### 4.4.2. Combination Forecasting Model Based on Shapley Value Method

According to the prediction results of three single prediction model methods, the total error ratio of Shapley combination prediction is calculated as *E*=1/3(*E*_1_+*E*_1_+*E*3). According to Shapley value combination forecasting method, the error ratios of all subsets of the three forecasting methods are *E*{1}, *E*{2}, *E*{3}, *E*{1,2}, *E*{1,3}, *E*{2,3}, *E*{1,2,3}, respectively, and their numerical values are the average values of vector error ratios included in the subsets. According to the Shapley value error distribution formula, the Shapley values of the three forecasting methods are *E*_1_^/^*E*_2_^/^, and *E*_2_^/^, respectively. Then, according to Shapley weight calculation formula, the corresponding weight is obtained.

#### 4.4.3. Combined Forecasting Model Based on IOWA Operator

According to the prediction accuracy of the ARIMA model, grey Verhulst model, and BP neural network model in each period, obtain the prediction error information matrix of third-order-induced ordered weighted arithmetic average, and the optimal combination weight can be obtained by solving it. Substituting the prediction error information matrix into the combination prediction model based on IOWA operator, the combination prediction model based on IOWA operator for aircraft failure rate prediction is constructed.

The weight coefficients of the three combined prediction models solved by LINGO software are shown in Table 2.

## 5. Comparison and Analysis of Prediction Results of Each Models

**Figure 8:**
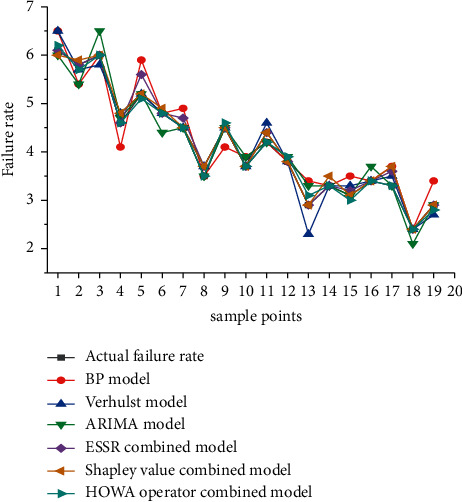
Comparison between the predicted value of input training failure rate and the actual value.

Due to the randomness and strong nonlinearity of aircraft failure rate, the evaluation of its prediction effect is different from the traditional methods adopted by other objects. The evaluation of aircraft failure rate prediction model can not only be conducted from one aspect or one index but also needs to be combined with multiple aspects. The constructed prediction model is used to systematically predict the training input of 1–20 groups of samples, and the prediction comparison of the input samples is shown in Figure 8. At the same time, the model accuracy and performance of various predicted failure rates are analyzed and evaluated by 7 evaluation indexes. Calculate the corresponding Mean Absolute Percentage Error (MAPE), Root Mean Square Error (RMSE), Mean Absolute Error (MAE), Index of Agreement (IA), Theil Inequality Coefficient (TIC), Equal Coefficient (EC), and Nash-Sutcliffe Efficiency coefficient (NSE), and obtain the input data model and error, as shown in Table 3.

From Figure 8, it can be seen that the predicted value of combined forecasting model of IOWA operator is in good agreement with the actual value of fault rate. At the same time, by comparing the data of each index in Table 3, it can be seen that the values of MAPE, MAE, TIC, and EC of ARIMA model are smaller in the three monomial models, while the values of RMSE of grey Verhulst model are the smallest, NSE deviates greatly from positive number 1, and the maximum values of IA, EC of the decision system using BP model, and the distribution of index comparison is irregular. In addition, from Table 3, it can be seen that each index of the combination model is better than that of the single model. At the same time, the combination forecasting model based on IOWA operator is the best one, followed by the combination forecasting model based on Shapley and ESSR method. The first three indexes of combination forecasting method based on IOWA operator are significantly lower than those of other methods, while IA, EC, and NSE are larger. Although the TIC value of combination forecasting method based on IOWA operator is not the minimum, it is close to the TIC of combination forecasting method based on Shale combination and ESSR method. Therefore, the combined forecasting model based on IOWA operator has the best effect than other models.

**Figure 9:**
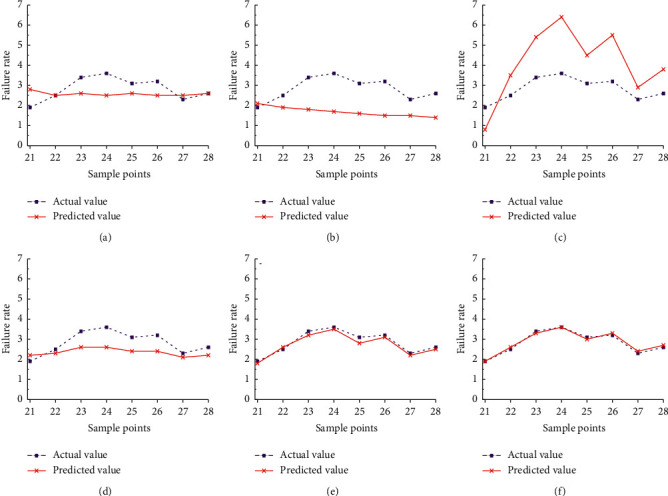
Comparison of predicted and actual failure rates of different models. (a) ARIMA model. (b) Verhulst model. (c) BP model. (d) ESSR combined model. (e) Shapley value combined model. (f) IOWA operator combined model.

In order to verify the validity of further model optimization, three single model prediction models (ARIMA model, grey Verhulst model, and BP neural network model) and three combination prediction models (combination forecast model of ESSR method, combination forecast model of Shapley value, and combination forecast model of IOWA operator) are used to verify the aircraft failure rate prediction for the last eight groups. The trend of the predicted value of actual failure rate corresponding to each model is shown in Figure 9.

From Figure 9, it can be seen that in various single prediction models, the deviation between the 21 and 28 sample points of ARIMA model and the actual failure rate value is large, and the grey Verhulst model also has certain deviation, and the fitting effect is unstable. At the same time, the deviation of prediction results of BP neural network model is larger, which is due to the defects of BP neural network model. The smaller the proportion of training samples is, the worse the generalization ability is. Among the three single models, ARIMA and Verhulst model's prediction effect is generally better than BP model's, but the overall prediction effect is average, which can only roughly predict the aircraft failure rates. The predicted values of aircraft failure rate obtained by three different combination models are similar to the actual values. Overall, the combination model has a high degree of fitting, and the deviation between the actual values and the predicted values is the smaller, which is better than the single model. The combined forecasting model can reduce the sensitivity to the poor single forecasting model. Although not every combined forecasting value is better than the best forecasting result of the single forecasting model, it must be better than the worst forecasting result. This shows that the combined forecasting model can effectively reduce the occurrence of large errors and improve the forecasting accuracy as a whole. The prediction result of the combined prediction model of the error sum of squares reciprocal method is inferior to that of the Shapley value method, and the prediction accuracy is not obviously improved compared with the single model, but it is even lower than its prediction accuracy. The combination forecasting model of IOWA operator method has a higher fitting degree than the combination forecasting model of the error sum of squares reciprocal method and the Shapley value method, and is closer to the actual failure rate. At the same time, the combination forecasting model of the IOWA operator method is better than the three single forecasting models.

Due to the randomness and strong nonlinearity of aircraft failure rate, its prediction effect evaluation is different from the traditional methods adopted by other objects. The prediction model of aircraft failure rate cannot be evaluated only from one aspect or one index but needs to be evaluated in combination with many aspects. Using the constructed prediction model, samples 1–20 are input into the system for prediction, and the accuracy and performance of various prediction failure rate models are analyzed and evaluated by seven evaluation indexes. Calculate the corresponding mean absolute percentage error (MAPE), root mean square error (RMSE), mean absolute error (MAE), index of agreement (IA), Theil inequality coefficient (TIC), equalization coefficient (EC), Nash-Sutcliffe efficiency coefficient (NSE), and get the input data model and error as shown in Table 3.

According to the comparison of the index data in Table 3, among the three single models, the MAPE, MAE, TIC, and EC values of the ARIMA model are smaller than those of grey Verhulst model, the RMSE value of grey Verhulst model is the smallest, but NSE deviates greatly from positive number 1, and the IA and EC values of BP model are the largest. Therefore, the distribution of each index is irregular. In addition, it can be seen from Table 3 that all indexes of the combined model are better than those of the single model. Meanwhile, the combined forecasting model based on the IOWA operator method is the best model, followed by the combined forecasting model based on the Shapley method and the error sum of squares reciprocal method. The first three indexes of combination forecasting model based on the IOWA operator method are obviously lower than other models, and its IA, EC and NSE are larger. Although the TIC value of the combination forecasting model based on IOWA operator is not the smallest, it is very close to the TIC value of the combination forecasting model based on the Shapley value method and the error sum of squares reciprocal method. Therefore, the combination forecasting model based on IOWA operator has the best effect compared with other models.

**Figure 10:**
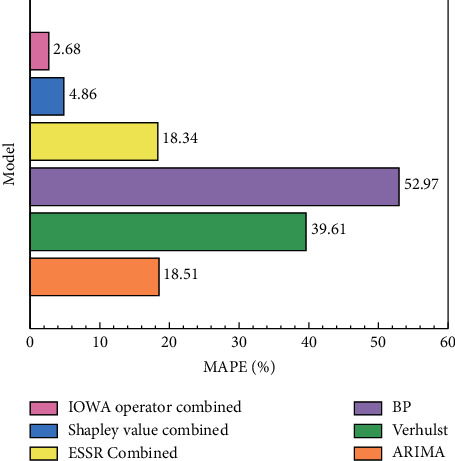
Comparison of MAPE for different models.

**Figure 11:**
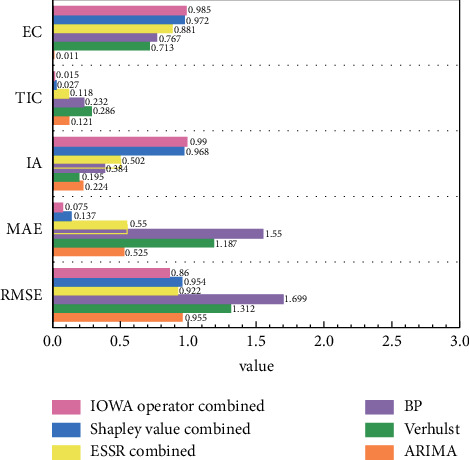
Comparison diagrams of different models EC, TIC, IA, MAE, and RMSE.

**Figure 12:**
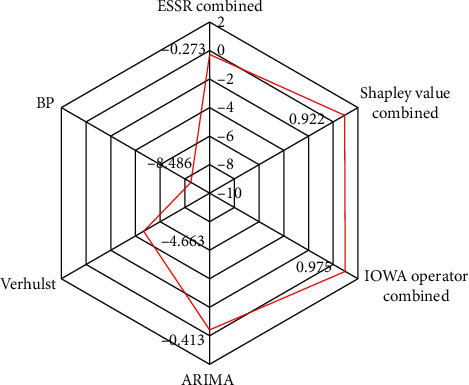
Comparison of NSE of different models.

In order to better evaluate the prediction models, we compared the error indices of the inspection data in different models. We selected the last 8 groups of inspection data samples to calculate the errors by using the single model and combined model, and obtained the corresponding MAPERMSE, MAE, IA, TIC, EC, and NSE when different models were adopted. The comparison results are shown in Figures 10–12.

The comparison of MAPE between the combined model and the single model in Figure 10 shows that the MAPE index of IOWA combined model is 2.68%, less than 10%, which is the smallest compared with other models, with a decrease of 44.8% (compared with Shapley combined model) to 94.9% (compared with BP model), indicating that IOWA combined model has stronger prediction ability. It can be seen from Figure 11 that the EC value and IA value of IOWA combined model for inspection data are 0.985 and 0.99, respectively, which are higher than those of other models, while TIC, MAE, and RMSE of IOWA combined model are relatively smaller than those of other models, with TIC = 0.015, MAE = 0.075 and RMSE = 0.86, respectively. The lower the values of these indexes, the higher the accuracy of the model. It can be seen from Figure 12 that the NSE index of BP model is −8.486, which deviates greatly from 1, while the NSE index of IOWA combination model for inspection data is 0.975, which is close to 1, which also indicates that the effect of IOWA combination model is better. Therefore, IOWA combination model among the three combination models proposed in this paper improves the performance and accuracy of aircraft failure rate prediction.

The above indicators only show part of the results of the prediction model performance. In order to evaluate the model more effectively, we analyze and study the comprehensive evaluation indicators of aircraft failure rate of prediction model in the inspection stage. By normalizing the above seven indicators, we get the expression C of the comprehensive evaluation index as shown below.

(9) $$\begin{array}{c} C_{i}=\frac{1}{n}\overset{n}{\underset{j=1}{\sum }}\frac{\min\left(E_{j}\right)}{E_{ij}}\text{,} \end{array}$$

**Figure 13:**
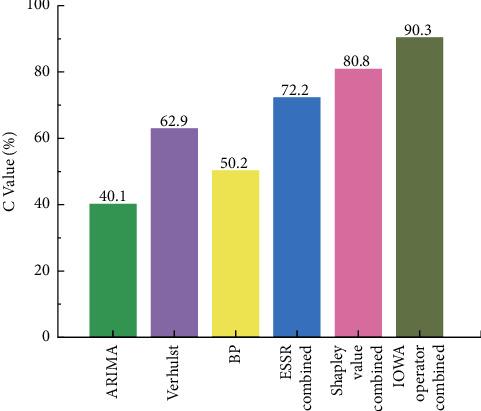
Comparison of comprehensive indexes between single and combined models.

*C*_*i*_ is the comprehensive evaluation index of the *i*-th prediction method, *i*=1,2,…, *M*, *E*_*ij*_ is the *j*-th indicator of the *i*-th method, *j*=1,2,…, *n*, and min(*E*_*j*_) is the minimum value of the *j*-th indicator in the *m*-th method. The higher the value of *C*, the better the prediction effect of the corresponding combined prediction model [43]. The seven indicator values for the eight sets of data after prediction were substituted into equation (9) to obtain the indicator C values for each method, as shown in Figure 13.

It can be seen from Figure 13 that the comprehensive evaluation index of each combined forecasting model is obviously higher than that of the three single forecasting models, indicating that the combined forecasting model can improve the forecasting accuracy of aircraft failure rate. Furthermore, the C of the combination forecasting model based on IOWA operator is 90.3%, which is obviously higher than other combination forecasting models. Through comparison, it can be seen that the IOWA operator model is better than the Shapley combination model as a whole, the Shapley combination model is better than the combination prediction model of the error sum of squares reciprocal method, and it is better than the single models, with higher performance, accuracy, and reliability. All analyses show that the combination forecasting model based on the IOWA operator is the best model, followed by the combination forecasting model based on the Shapley value and the combination forecasting model based on the error sum of squares reciprocal method.

At the same time, in order to verify the accuracy of the Iowa operator combination model, GM (1, 1) model [44], SVM model [45, 46] (Parameters Optimization of SVM Using RBF Kernel Function, setting parameter *γ*=10.023, *C*=32.121), entropy weight method combination model [47], and XGBoost model [48] (Determination of parameters by grid search method learning_rate = 0.05, max_depth = 4, subsample = 0.9, min_child_weight = 2, gamma = 0.5, colsample_bytree = 0.6) are used for comparison and analysis. The accuracy indexes of different models are shown in Table 4.

Table 4 provides a comprehensive comparison of the prediction accuracy indexes between the proposed model and the GM (1, 1) model, SVM model, entropy weight combined model, and XGBoost model. From Table 4, it can be seen that the accuracy of the IOWA operator combination model is better in seven prediction accuracy indexes, and the comprehensive evaluation index C is larger than other models, which can verify that the proposed IOWA model is better than other models. Obviously, the proposed combination model based on IOWA operator has good prediction performance. In addition, Pearson test is introduced to determine the fitting degree between the predicted model and the actual model. Pearson test can show the correlation between the actual value and the predicted value. The closer the correlation coefficient is to 1, the more linear the relationship between actual value and predicted value is. The closer the correlation coefficient is to 0, the smaller the correlation between the actual value and the predicted value [49].  Table 5 showed Pearson test values of the above models.

It can be concluded from Table 5 that the proposed combined prediction model method of Iowa operator has relatively high Pearson test correlation coefficient compared with the GM (1, 1) model, SVM model, entropy weight combination model, and XGBoost model. Therefore, it can be shown that the data correlation between the predicted value and the actual value of the proposed prediction method is stronger. The prediction accuracy of the proposed prediction method is higher than that of other models, and the predicted data value is closer to the actual value.

**Figure 14:**
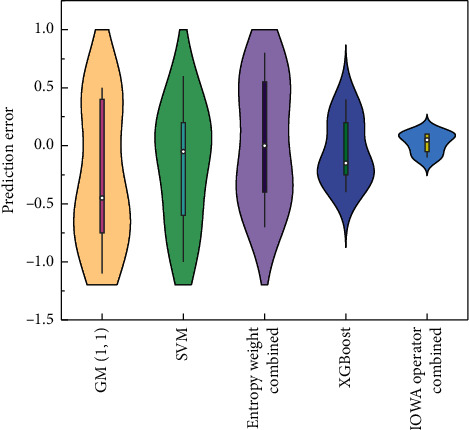
Forecasting errors of different models.

Violin diagrams are a collection of boxplots and nuclear density maps, which show percentiles of data through box-line thinking, while nuclear density maps are also used to show contour effects of data distribution. Larger contours mean more data are concentrated there, or vice versa, less data are available there. It is very suitable for judging and analyzing forecast error.  Figure 14 shows the violin diagram of forecast error of each prediction model. It can be clearly seen that the combined forecasting model of IOWA operator proposed has advantages in forecasting error, followed by XGBoost combined forecasting model. Compared with other models, the forecasting error is smaller and the forecasting accuracy is higher. It can effectively reduce the forecast error and is a stable and reasonable forecast method for aircraft failure rate.

**Figure 15:**
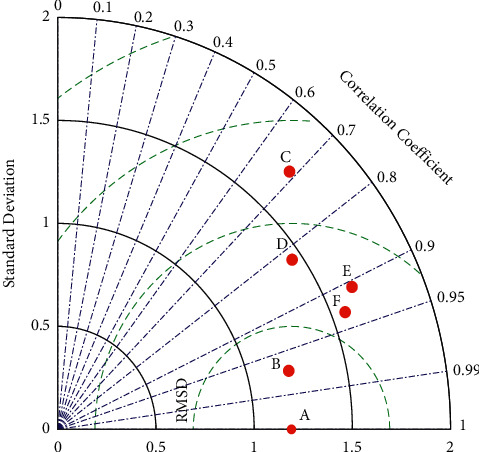
Taylor diagram of forecast results (A: actual value; B: IOWA operator combination model; C: GM (1, 1) model; D: SVM model; E: Combination model of entropy weight method; F: XGBoost model).

To describe the predictive results of different prediction models, Taylor charts are introduced. As shown in Figure 15, horizontal and vertical coordinates represent standard deviations, sector curves represent correlation coefficients, and dashed lines represent root mean square deviation (RMSD). As can be seen from Figure 15, Point B is closer to Point A, so the correlation coefficient of the IOWA operator combination model is larger than that of other contrast models, and the predicted value of the prediction model fits the observed (actual) value better. In addition, the combined model prediction model of IOWA operator has smaller RMSD and has similar standard deviation with the observed (actual) value. Overall, the combined model of IOWA operator has better performance.

**Figure 16:**
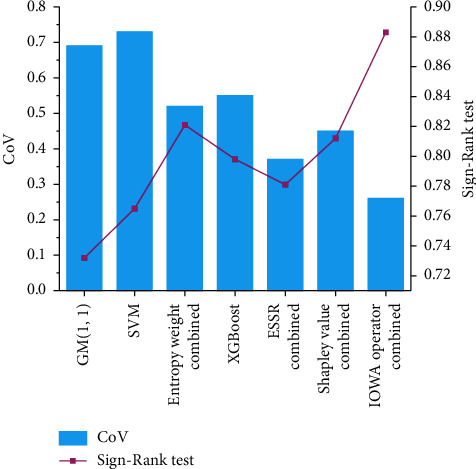
Comparison of CoV and ign-rank test for different models.

To further verify the uncertainty of different models, coefficient of variance is used to verify. The coefficient of variation is a statistical indicator to measure the degree of dispersion and variation of each observed value. The ratio of standard deviation to mean is taken as the coefficient of variation (CoV) [50], and the statistical significance of prediction model is tested. The Wilcoxon Sign-Rank test [51] is introduced. The Wilcoxon Sign-Rank test results between the predicted value and the actual value of each prediction model are obtained. The CoV and Wilcoxon Sign-Rank tests of different models are shown in Figure 16.

From Figure 16, it can be seen that the combined forecasting model method of IOWA operator has smaller coefficient of variation than the GM (1, 1) model, SVM model, Entropy Weight Combination model, XGBoost model, combination forecasting model of ESSR method, and Shapley value combination forecasting model. In terms of uncertainty quantification, the combined forecasting model of IOWA operator provides more satisfactory CoV than other six model methods, indicating that the influence of uncertainty of this model is smaller. It can also be seen that the Wilcoxon Sign-Rank test value of the combined forecasting model of IOWA operator is larger than that of other comparative forecasting models. Compared with other forecasting models, the median difference between the forecasting value and the actual value is small, which can meet the actual needs of aircraft failure rate forecasting.

The program execution time is one of the indexes in modeling and calculation processing, which determines the training and prediction time of the model. The execution time of IOWA operator combination model is 0.35 s, which compares with 0.72 s for the GM (1, 1) model, 1.89 s for SVM model, 0.63 s for Entropy Weight Combination model, 1.25 s for XGBoost model, 0.98 s for combination forecasting model of ESSR method, and 0.56 s for Shapley value combination forecasting model, and the time taken is shorter. It shows that IOWA operator combination model has a low computational time complexity, can achieve a faster learning process and a satisfactory and acceptable prediction effect.

## 6. Discussion

Compared with the single prediction model, the combination prediction model has obvious advantages, which can effectively improve the prediction accuracy of aircraft failure rate. The three combination prediction models proposed in this paper can ensure the accuracy of aircraft failure rate prediction, so the combination model is more practical in the field of aircraft failure rate prediction. At the same time, the evaluation indexes include MAPE, RMSE, MAE, and TIC. The combined prediction model based on IOWA operator are smaller than those of ARIMA, grey Verhuls, and BP models. The error sum of squares reciprocal combined model and Shapley combined model are smaller. At the same time, its IA, EC and NSE indexes are improved. It shows that the increase in the prediction accuracy is related to the weight coefficient of the combined model, and the selection of appropriate weights can effectively improve the prediction accuracy of the model. Because the aircraft failure rate has the characteristics of strong random accidental interference, poor information, and nonlinear data, ARIMA, grey Verhulst, BP model, and IOWA operator combination model are ideal.

The ARIMA forecasting single model is simple and suitable for endogenous variables. However, it also has the shortcomings of requiring the time series data to be stable and unable to capture the nonlinear relationship. The grey Verhulst single model is not a kind of strict method, which avoids the analysis of the system structure, and directly builds the saturation growth model of load by cumulating the original data. Its prediction requires less original information, and the calculation process is simple. It is suitable for the prediction of saturated load with the lack of original data and the load changing according to the *S*-shaped curve, and it is suitable for the prediction of the failure rate of small sample aircraft. When the number of samples is small, the prediction results of BP single forecasting model will be inaccurate, which directly affects the generalization ability of neural network, so it is more suitable for occasions with large amount of data. Solving the weight coefficient by the error sum of squares reciprocal method has the advantages of simple calculation, satisfying the nonnegative requirement of fusion degree, and determining the fusion degree of a single model in the combined model according to the error sum of squares. However, its prediction accuracy is not high, so it is only suitable for forecasting occasions with low accuracy. The Shapley value method can allocate the fusion degree of each model according to the contribution of the error sum of squares of the combined model according to each single forecasting model, which effectively reduces the error of the model and improves its accuracy. It is also suitable for the prediction occasions with low accuracy. The IOWA operator method obtains the weight coefficients of each model according to the minimum criterion for sum of squares of error, which effectively reduces the influence of errors, and has high prediction accuracy. The proposed combination forecasting model based on IOWA operator can reduce MAPE, RMSE, MAE, and TIC indexes of GM (1, 1) model, SVM model and combination model of Entropy Weight Method, XGBoost model, 8.23% of MAPE, 0.74% of TIC, 2.38% of RMSE and 2.03 of MAE compared with XGBoost model and GM (1, 1) model. The IA, EC, NSE, and C composite indices of the combined forecasting model are improved compared with other models. Compared with the GM (1, 1) model, the IA and EC of the combined forecasting model are increased by 0.34 and 0.2, respectively, while the NSE and C composite indices are increased by 0.73 and 33.4%, respectively. At the same time, the combination forecasting Pearson correlation coefficient based on HOWA operator reaches 0.972, which is also at a high level with other models, indicating that the model is more effective and stable and reliable.

The prediction errors of IOWA operator with the GM (1, 1) model, SVM model, entropy weight method combined model, and XGBoost model are shown by violin plots, and their prediction errors are smaller. The combined model of IOWA operator is also known to have smaller RMSD by comparing Taylor plots, but the correlation coefficient is larger than other comparative models. Therefore, the established prediction model has high prediction accuracy and can correctly reflect the prediction of failure rate. Meanwhile, the GM (1, 1) model, SVM model, entropy weight combination model, XGBoost model, ESSR combination prediction model, and Shapley value combination prediction model have smaller coefficient of variation and good certainty, which is 0.47 lower than that of the SVM model. Meanwhile, the Sign-Rank test value of the IOWA operator combination model is also larger than that of other comparative prediction models, and the ESSR method. The combined prediction model has higher indicator and Shapley values. The Sign-Rank test value of the IOWA operator combination model is 0.15 higher than the GM (1, 1) model, which is a better prediction model. Statistically, the effectiveness of the combined model was verified.

By comparing the results obtained in this paper, it can be concluded that the combination model can improve the prediction accuracy, but the improvement range of the estimation accuracy of different combination models is different. This is mainly due to the influence of several aspects, such as the selection of the single model, the determination of the weighting coefficient of the combination model, and the practical application of the model.

## 7. Conclusion

The ARIMA model, grey Verhulst model, and BP neural network model are selected as single models, and three combined prediction models are constructed based on them, which improves the prediction accuracy and optimizes the prediction effect. The combination model can comprehensively utilize the information of each single model, and comprehensively consider the advantages of each single prediction model. The prediction error of the single prediction model is dispersed to reduce the occurrence of large errors. Therefore, compared with the single prediction model, the prediction accuracy of the combination prediction model is significantly improved. At the same time, the combined model based on IOWA operator has high prediction accuracy, higher stability, and stronger applicability, which can meet the demand of aircraft failure rate prediction and provide some ideas for aircraft failure rate prediction.

The combined model prediction model proposed in this paper fully demonstrates the excellent performance of the combined prediction model through performance indicators, error analysis, Pearson test, violin chart, Taylor chart, and other aspects, and verifies the prediction effect. The combined model is not only suitable for the prediction of aircraft failure rate but also for other equipment indicators or parameters with time series characteristics, such as aviation material consumption, flight safety accident rate, aviation equipment integrity rate, etc. It provides a scientific method and means for equipment support prediction. At the same time, it also helps to improve the performance of online health state estimation methods for other key functional systems and core key components of aircraft, and supports the breakthrough of key prediction technologies in aerospace, weapons and ships, intelligent equipment, and other fields, which has practical reference significance and reference value.

The combined model proposed in this paper also has some shortcomings. It only uses historical data to predict the future aircraft failure rate. However, in fact, the aircraft system is a complex system, and the failure rate is affected by many other external input factors, such as ambient temperature, ambient humidity, flight hours, and support capability, which lead to high nonlinearity and uncertainty of the aircraft failure rate. Therefore, all these factors should be considered when establishing a multi-input prediction model, and carrying out multi-source data mining and considering the influence of multi-state interleaving to further improve the prediction accuracy of aircraft failure rate. At the same time, in the future, we will further explore the new application of combination model and develop complex combination forecasting model to improve the overall forecasting quality and effect.

## Figures and Tables

**Table 1:** Aircraft failure rate prediction method.

Failure rate prediction method	Single model	Statistical model	Regression analysis [1], time series [2, 3], mathematical statistics [4], Weibull distribution statistics [5], Bayesian [6]
		Grey model	GM (1, 1) [7–9], Verhulst [10]
		Machine learning model	Artificial neural network (ANN) [11], BP neural network [12–14], generalized regression neural network (GRNN) [15], support vector machine (SVM) [16], least squares support vector machine (LS-SVM) [17], random forest [18]
		Deep learning model	Long short-term memory (LSTM) [19], convolutional neural network (CNN) [20]
	Combined model	Model-based combination forecasting	Grey neural network-fuzzy recognition [21], artificial neural network and genetics [22], MLR-GM (1, N)-PLS-BP-SVM [23], SVR- multiple regression-principal component analysis [24], ARMA-BP [25], grey model combination [26, 27]
		Method-based combination model	Holt-winters seasonal model [28], neural network residual correction AR [29], artificial neural network Weibull regression [30], Weibull-based generalized renewal process (WGRP) [31], sparse direct support vector machine regression [32], generalized weighting least-squares combination [33]
		Integrated combination model based on decomposition	Empirical mode decomposition (EMD) and LS-SVM combination [34], correlation vector EMD and GMDH combination [35], EMD and RVM-GM combination [36], CEEMD and combined model [37]

**Table 2:** Weight coefficient of combined forecasting model.

Combined forecasting model	Weight coefficient		
	*ω*_1_	*ω*_2_	*ω*_3_
ESSR combined model	0.39	0.48	0.13
Shapley value combined model	0.31	0.35	0.34
IOWA operator combined model	0.43	0.22	0.35

**Table 3:** Input data model and error data table.

Model		MAPE	RMSE	MAE	IA	TIC	EC	NSE
ARIMA		17.34	1.223	0.235	0.224	0.223	0.109	-1.335
Verhuls		22.31	0.908	1.118	0.206	0.312	0.625	-2.109
BP		24.54	1.221	1.223	0.403	0.156	0.798	-1.478
Combined	ESSR method	10.23	0.712	0.233	0.732	0.213	0.831	0.255
	Shapley value method	9.43	0.109	0.156	0.897	0.132	0.912	0.836
	IOWA operator method	5.42	0.101	0.089	0.987	0.134	0.981	0.934

**Table 4:** Accuracy data table of different models.

Accuracy indexes	Models compared				
	GM (1, 1)	SVM	Entropy weight method combination	XGBoost	IOWA operator combination
MAPE	15.25	12.13	10.91	8.92	2.68
RMSE	1.32	3.24	0.98	1.12	0.86
MAE	2.112	1.097	1.009	0.976	0.075
IA	0.65	0.88	0.86	0.94	0.99
TIC	0.509	0.399	1.023	0.856	0.015
EC	0.786	0.887	0.901	0.809	0.985
NSE	0.245	0.876	0.793	0.853	0.975
C (comprehensive)	56.9	87.6	78.9	88.9	90.3

**Table 5:** Pearson correlation coefficient.

Forecasting model	Pearson correlation coefficient
IOWA operator combination	0.972
GM (1, 1)	0.686
SVM	0.823
Entropy weight method combination	0.908
XGBoost	0.932

## Data Availability

The maintenance data used to support the findings of this study have not been made available because sharing the data might compromise data privacy.
